# Reliability of radiotherapist assessment for radiation induced skin-toxicity: investigational study

**DOI:** 10.3389/fonc.2026.1882432

**Published:** 2026-08-10

**Authors:** Lilian Doerwald-Munoz, Joseph Hayward, Fahad Alghannam, Qiyin Fang, Mohamed Hisham Aref, Ramy Abdlaty

**Affiliations:** 1 Juravinski Cancer Centre, Hamilton Health Sciences, Hamilton, ON, Canada; 2 Microelectronics and Semiconductors Institute, King Abdulaziz City for Science and Technology (KACST), Riyadh, Saudi Arabia; 3 Engineering Physics, McMaster University, Hamilton, ON, Canada; 4 Egyptian Armed Forces, Cairo, Egypt; 5 Biomedical Engineering, Military Technical College, Cairo, Egypt

**Keywords:** dermatology, visual diagnosis, skin erythema, ionizing radioactivity, radiation therapy, skin toxicity, skin redness, digital imaging in medicine

## Abstract

**Purpose:**

Enhancing the quality of radiotherapy for skin cancer requires systematic evaluation of radiation-induced skin toxicity, particularly acute toxicity, which affects ~90% of patients receiving ionizing radiation. This study assessed the reliability of a newly developed visual grading tool, the Clinician Erythema Assessment–Radiation Therapy (CEA-RT), designed to help radiation clinicians evaluate early radiation dermatitis.

**Methods and materials:**

The CEA-RT tool utilizes a 5-point scale. Its reliability was evaluated by analyzing intra-rater and inter-rater agreement among clinicians, as well as its concordance with an expert reference standard against a reference standard. The assessment was conducted using digital images.

**Results:**

The study’s results showed high intra-rater reliability, with 98% of scores falling within ±1 grade and consistent grading for regions of interest (ROI). However, inter-rater agreement was only fair, indicating variability among different clinicians. Concordance with an expert reference standard against a reference standard was moderate, with 25–30% of therapists achieving high concordance and over 50% showing moderate agreement.

**Conclusions:**

The findings support the CEA-RT scale as a reliable tool for individual use. However, study limitations included variable time gaps between sessions, recall bias, fatigue, and reliance on digital images. The results emphasize the need for additional training, *in vivo* validation, and inclusion of diverse skin types to improve overall accuracy and applicability.

## 1 Introduction

Occupational skin cancers constitute a substantial proportion of all reported occupational diseases (1). The persistent increase in skin cancer incidence over recent decades has established it as a major global public health priority. Epidemiologically, skin cancer is the most prevalent malignancy globally, representing approximately more than 40% of all cancer diagnoses (2). Within the United States, its annual incidence exceeds that of all other cancers combined, incurring estimated treatment costs of $8.1 billion (3). Solar ultraviolet radiation (UVR) is the paramount risk factor (4). According to joint estimates by two international organizations such as the World Health Organization (WHO) and the International Labor Organization (ILO), occupational exposure to UVR affects approximately 1.6 billion individuals worldwide, accounting for 28.4% of the global working-age population (5).

Skin cancer (SC) administration employs a range of therapeutic modalities, with surgery and radiotherapy (RT) being the most frequently applied (6). Surgical intervention is often the primary and preferred treatment when clinically indicated (7). However, for senior patients, surgery may be ineligible due to comorbidities that pose a life-threatening risk or the potential for a significant negative impact on quality of life (8). Consequently, a substantial proportion of geriatric SC patients are referred for RT as a prime treatment alternative (9).

Despite its worth, RT has some limitations, as it can induce a range of adverse effects, most notably radiation-induced skin toxicity (10). This toxicity is a highly common sequela, affecting approximately 9 out of 10 SC patients undergoing RT treatment (11). The severity of these reactions exists on a spectrum from mild to severe and typically manifests in a predictable temporal sequence (12):

- Primitive Erythema: The most immediate skin reaction is inflammation (erythema), which can arise within hours’ post-irradiation. In some cases, however, its onset may be delayed for up to two weeks following the initiation of RT (13).
- Dry and Moist Desquamation: After approximately three weeks of radiation, damage to the basal layer of the epidermis results in painful epidermal necrosis. This clinically presents as desquamation (peeling), which can be either dry or moist (involving serous exudate) (14).
- Ischemia and Necrosis: Within three months of treatment initiation, cutaneous ischemia and frank necrosis may occur as a consequence of vascular damage and compromised tissue perfusion (15).
- Postponed Effects: Delayed and often chronic skin changes, including telangiectasia (dermal capillary dilation), fibrosis, and severe dermal necrosis, can emerge approximately twelve months after irradiation (16).

**Figure 1:**
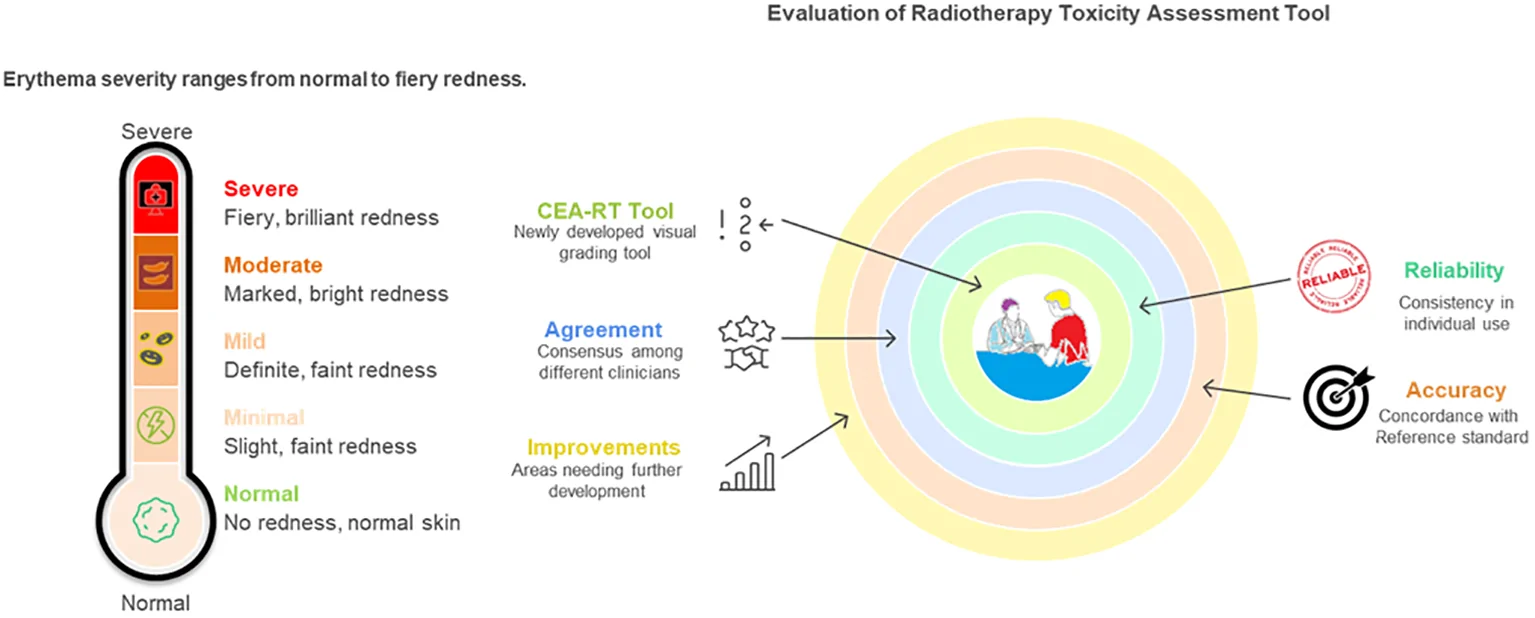
The left panel illustrates the varying symptoms of erythema severity, ranging from faint redness of the skin to intense erythematous reactions of the epidermal tissue. The right panel presents a visual summary of the study, outlining the methodology (development of the CEA-RT tool), key findings (inter-clinician agreement), and the discussion and conclusion (the required improvements to achieve higher reliability and accuracy).

As previously noted, cutaneous erythema is a prevalent sequela of radiation therapy, manifesting as an inflammatory response characterized by a pronounced reddening of the skin (17) (18) (19),, as shown in **Figure 1**. This erythema represents a visible indicator of pathophysiological impairment to the epidermal basal layer, which subsequently triggers a localized vasodilation response, increasing dermal blood flow and volume (20). While numerous validated visual grading scales exist for radiation induced dermatitis, the validity of these instruments for quantifying early-stage radiation-induced skin inflammation remains inadequately explored (21). The existing RTOG and CTCAE grade for erythema, edema, and desquamation jointly are not designed to resolve fine gradations of early, isolated erythema. Moreover, CTCAE-graded dermatitis has also been shown to correlate only weakly with patients’ own symptom reports (22). CEA-RT is intended as a complementary, erythema-specific instrument for this earlier phase, not a replacement for composite toxicity grading. The primary objective of this study was to systematically evaluate the reliability of a modified clinician erythema assessment tool, adapted for the specific context of radiation oncology. This revised technique, designated the CEA-RT, is a five-level ordinal scale developed to standardize the grading of erythema associated with early radiation dermatitis by clinical practitioners, including radiation therapists and oncologists. A critical refinement of the CEA-RT is its utilization of descriptive terminology that aligns precisely with the specialized lexicon of radiation oncology, thereby enhancing its clinical applicability and face validity within the field as displayed in **Figure 1**. CEA-RT adapts the Clinician’s Erythema Assessment, a five-point ordinal erythema scale originally validated for facial erythema in rosacea (23) and subsequently adapted for other dermatoses (24), substituting radiation-oncology-specific descriptive terminology. To our knowledge, this is the first reliability evaluation of this adapted instrument specifically for radiation-induced erythema, not a claim of a new assessment method.

Reliable and reproducible grading of radiation-induced erythema is not merely an academic exercise; it directly informs clinical decision-making throughout the RT course. Timely and accurate grading enables evidence-based implementation of prophylactic and therapeutic interventions — including emollient and moisturizer regimens, corticosteroid creams, barrier dressings, and, in severe cases, treatment interruption or replanning — at the appropriate severity threshold. Recent systematic reviews and evidence syntheses have identified the absence of standardized grading as a barrier to the implementation of evidence-based dermatitis management pathways, as treatment thresholds and outcome assessments across studies are not directly comparable when different scales are applied inconsistently (25) (26) (27),,. The development and reliable application of a standardized, radiation-specific erythema scale such as CEA-RT therefore have direct implications for improving consistency in clinical care pathways, enhancing the comparability of clinical trial outcomes, and ultimately reducing patient morbidity associated with radiation-induced skin toxicity.

## 2 Materials and methods

A cohort of twenty therapists, with clinical experience ranging from 4 to 35 years, was recruited for this study. The distribution of years of experience was as follows: 40% (n=8) had 4–10 years, 30% (n=6) had 11–20 years, and 15% (n=3) each had 21–30 and 31–35 years. The study cohort comprised 80% (n=16) female and 20% (n=4) male participants. For context, the demographic composition of all therapists (n=90) at the JCC at the time of the study was 76% female and 24% male, with a range of clinical experience from 1 to 35 years.

### 2.1 Clinical procedure and study participants

This single-center study was conducted to evaluate the reliability of the CEA-RT, a five-level visual assessment scale revised for use in radiation therapy, in grading the severity of radiation-induced erythema. All participants provided written informed consent prior to enrollment. Therapists applied the CEA-RT technique to grade erythematous reaction in digital images obtained from nine patients (aged 56–88 years) treated at the Juravinski Cancer Centre (JCC) in 2015, 2016, and 2017. A brief information for the recruited patients and their treatment characteristics and demographics is included in **Table 1**. Treatments were delivered with curative intent using either electron beams (6–12 MeV) or orthovoltage x-rays (65–130 kVp). None of the patients had received prior RT to the same anatomical site. The prescribed radiation doses varied between approximately 31 Gy and 50 Gy, delivered over treatment periods of 2, 3, or 4 weeks, with five fractions administered per week. Irradiation was administered to several anatomical sites, including the extremities, cervical region, face, forehead, and pinna. The study protocol was reviewed and approved by the institutional research ethics board. All patients were Caucasian (four males and five female) and had histologically confirmed either early-stage basal cell carcinoma (BCC) or squamous cell carcinoma (SCC) of the skin. One female patient was excluded from the analysis accounting for the original description due to the unavailability of photographs. **Table 2** describes the numbers of the participating patients in the study.

**Table 1:** Patients demographics for radiotherapist assessment for radiation induced skin-toxicity.

Patient	Gender	Age	Histology	Radiation	Energy	Dose (cGY)	Frac.	Body cites
P01	Male	84	SCC	Electrons	6 MeV	3160	10	Rt neck
P02	Female	82	SCC	Electrons	6 MeV	5000	20	Lt leg
P03	Female	88	SCC	Electrons	9 MeV	5000	20	Lt leg
P04	Female	56	BCC	X-rays	65 kVp	5000	20	Lt forearm
P05	Male	68	BCC	X-rays	65 kVp	4250	10	Lt check
P06	Male	85	BCC	Electrons	12 MeV	4250	10	Rt ear
P07	Male	75	SCC	X-rays	130 kVp	4250	10	Lt cheek
P08	Female	79	BCC	X-rays	65 kVp	4725	15	Forehead
P09	Female	68	SCC	Electrons	6 MeV	4720	15	Lt leg

**Table 2:** The number of the screened and the final participating patients in the current study.

Category	Number of patients
Patients initially recruited	9
Excluded patients (Photographs unavailable)	1 (female)
Patients contributing to ROI image set	8

### 2.2 Image acquisition

Digital skin images were acquired from a previously approved, consented clinical study involving Caucasian patients undergoing radiation therapy. Longitudinal image acquisition was performed throughout the treatment course to document the temporal progression of radiation-induced skin erythema.

Image acquisition was conducted using a high-definition digital camera (HYUNDAI-HDMI-768) equipped with a 16-megapixel image sensor, an objective lens (F/3.2, f = 7.5 mm), and a 3-inch LCD display for field-of-view (FOV) adjustment. The camera measured 110 × 58 × 50 mm and weighed 240 g. To minimize image variability attributable to external factors, all photographs were acquired under standardized imaging conditions, including consistent illumination and camera positioning (~25–30 cm from the patient), thereby ensuring uniform image quality and facilitating unbiased visual assessment.

For the observer evaluation, all images were anonymized and presented in a randomized sequence. Randomization was implemented to minimize recall and expectation bias associated with the known chronological progression of radiation-induced erythema during treatment.

### 2.3 Study design

**Figure 2:**
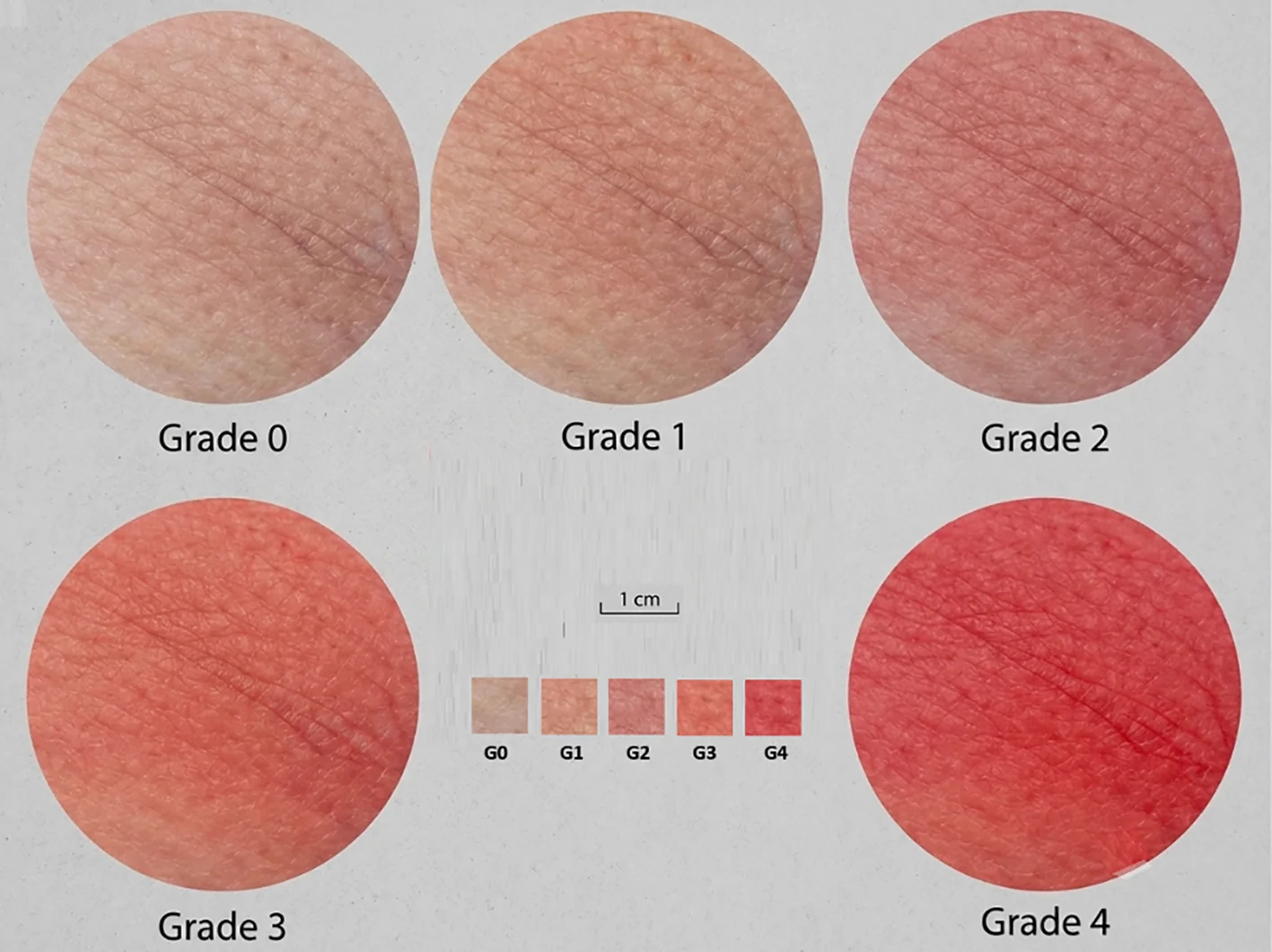
Reference diagram for the Clinician Erythema Assessment for Radiation Therapy (CEA-RT). This tool provides a standardized 5-point ordinal scale and corresponding clinical photographs to aid practitioners in consistently grading the severity of cutaneous erythema resulting from radiotherapy.

The study comprised three phases: one training session followed by two independent grading sessions. Training session: Radiation therapists were familiarized with the CEA-RT technique via a slide-based presentation. Sample images demonstrating erythema across the five CEA-RT levels were exhibited in **Figure 2**. The figure served as the photonumeric calibration reference used during the training session, consistent with the calibration approach used for the parent CEA instrument; the subsequent group consensus exercise confirmed uniform interpretation of each grade prior to independent scoring. A collaborative grading exercise was then conducted, during which participants assigned erythema grades as a group to achieve consensus and ensure grading consistency.

Grading sessions: Following training, each RT independently assessed digital images in two separate sessions. The same set of images was presented in identical order during both sessions, with a minimum interval of three hours between assessments to reduce recall bias. A methodological limitation of the current design is that the same images were presented in identical order across both grading sessions. Although a minimum inter-session interval of three hours was enforced to reduce recall, this interval may be insufficient to fully eliminate memory of previously assigned grades, particularly for experienced clinicians who may develop systematic grading patterns. Future designs should employ randomized image sequencing across sessions, increase the inter-session interval to at least one week, or intersperse distractor images between sessions to minimize this recall bias.

Two sets of images were evaluated in each grading session:

v. Field-of-view (FOV) images: 60 images from six patients, each showing the full treatment region. A circular region of interest (ROI) measuring 2 cm in diameter was marked on each image.
vi. ROI-only images: 98 images from eight patients, each displaying only the isolated ROI without the surrounding FOV.

The reasons for the difference in patient numbers between the FOV and ROI image sets — specifically that FOV images were available for a subset of six patients whose photographic acquisitions included the full treatment field. The summary of the included image sets is stated in **Table 3**. Patients #5, #7 and #8 were included in the ROI image-set but not included in the FOV image set because the images were of inferior quality (i.e. too dark, pixelated, blurry, etc.). Image quality was relevant for the FOV image-set since poor quality images would hinder the therapists’ ability to interpret the images clinically and erythema features with clarity.

**Table 3:** Summary of the involved FOV and ROI image sets which were included in the study.

Category	Number of patients
Patients contributing to FOV image set	6
Total FOV images analyzed	60
Patients contributing to ROI image set	8
Total ROI images analyzed	98

**Figure 3:**
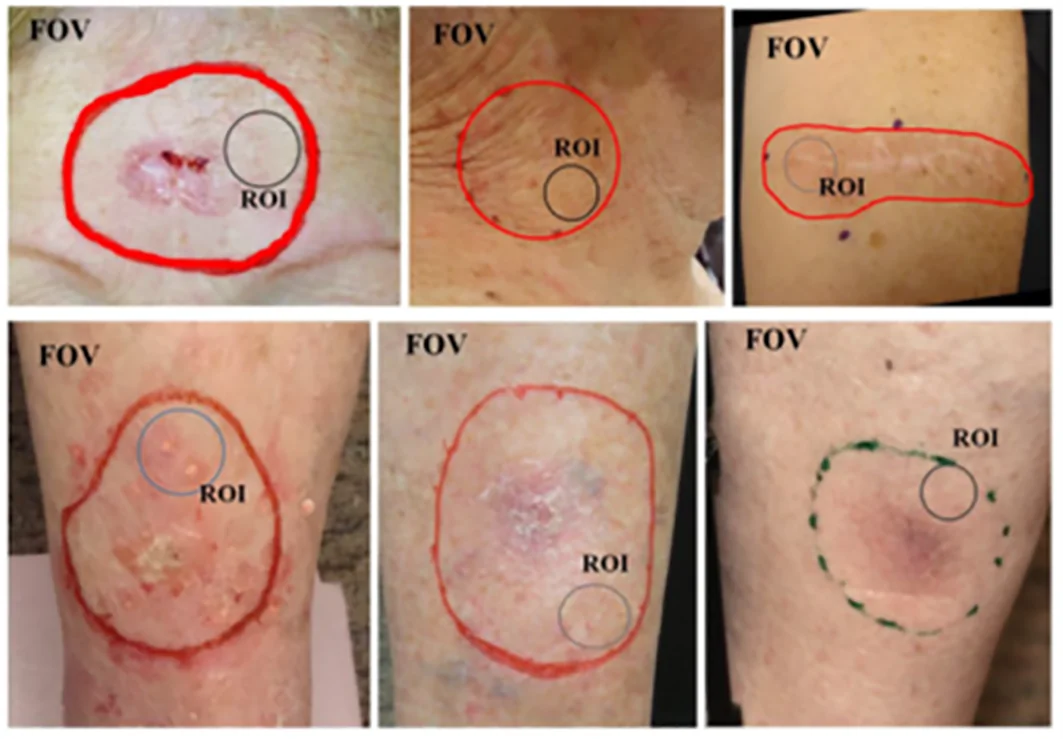
Six sample images are presented, each depicting the full treatment field or field of view (FOV). Within each FOV, a 2-cm circular region of interest (ROI) was delineated for erythema grading.

Therapists were directed to confine their erythema assessment solely to the ROI. For FOV images, the irradiated field was outlined, enabling comparison of irradiated and non-irradiated areas to support grading accuracy as displayed in **Figure 3**. A recognized methodological limitation inherent to skin-cancer RT studies is the potential for visual overlap between perilesional radiation-induced erythema and tumor-surface changes including crusting, regression, desquamation, and residual pigmentation within the irradiated field. In the present study, graders were specifically instructed to confine their assessment to perilesional skin erythema within the delineated ROI and to exclude direct tumor-surface changes. Nevertheless, the degree to which individual graders uniformly applied this distinction cannot be confirmed retrospectively, and residual ambiguity in grading decisions attributable to this overlap cannot be excluded. Future prospective studies should provide explicit written grading guidance and conduct inter-rater calibration exercises specifically addressing the tumor-field grading boundary.

All images were displayed on identical monitors under the same environmental conditions. The Participant radiotherapists were equipped with the necessary tools for assessment: the CEA-RT scale and a standardized scoring form. The reliability of the CEA-RT scale was evaluated by examining both intra-rater variability (consistency of repeated scores by the same therapist) and inter-rater variability (consistency among different therapists). Grading accuracy was determined by comparing each therapist’s scores, as well as pooled group scores, against a single-expert reference standard.

The single-expert reference standard was defined as the scores assigned by the study instructor—a senior radiation therapist with extensive expertise in evaluating radiation-induced erythema in both patients and photographic images using the CEA-RT scale.

## 3 Data analysis

This study evaluated the consistency and concordance with an expert reference standard of therapists’ grading of erythema using the CEA-RT scale on two image sets: field of view (FOV) and region of interest (ROI). Ordinal data were collected using a five-point severity scale, where 0 indicated normal skin status, 1 minimal erythema, 2 mild erythema, 3 moderate erythema, and 4 severe erythema. Because the scores represent attribute data, statistical analysis was performed using the Attribute Agreement Analysis module in Minitab^®^ 18 Statistical Software. This analysis assessed intra-rater reliability (within rater), inter-rater reliability (between raters), and agreement with a gold standard (concordance with an expert reference standard). The following statistical methods were applied. Unlike RTOG, which grades a composite of erythema, edema, and desquamation jointly, CEA-RT isolates erythema intensity as a single ordinal dimension, enabling finer discrimination during the early toxicity phase when desquamation and edema may not yet be present.

### 3.1 Absolute agreement

Absolute agreement denotes a statistical approach that evaluates the exact concordance between raters, instruments, or methods in their assessment of the same subject or item, emphasizing precise matching rather than mere correlation. It is a fundamental metric in reliability and agreement studies, with critical applications in domains such as clinical research, psychology, and quality control. For our study, each therapist graded all images twice, allowing intra-rater consistency to be quantified as the percentage of identical ratings across both sessions. Agreement was categorized as follows:<50% = poor, 50–70% = good, ≥70% = excellent (28). This method provides a straightforward measure of inter- or intra-rater reliability by calculating the proportion of cases in which identical scores or classifications are assigned (29). The computation is defined by the following Equation 1:

(1) $$Absolute\;Agreement\left(\%\right)=\frac{Number\;of\;exact\;agreements}{Total\;number\;of\;ratings}*100$$

Where:

- Number of exact agreements = the count of items/observations where raters provided identical ratings.
- Total number of ratings = the total number of items assessed across raters or sessions.

### 3.2 Fleiss’ kappa

Fleiss’ kappa is a statistical measure of inter−rater agreement for categorical data when there are more than two raters. It extends Cohen’s kappa beyond the two−rater case and adjusts for the amount of agreement expected by chance (30). In our case we have multiple radiotherapists with various years of experience. To evaluate the statistical significance of the inter-rater agreement, Fleiss’ kappa was calculated with an alpha level of 0.05. The hypotheses tested were:

H0: Agreement is attributable to chance.
H1: Agreement is greater than chance.

Kappa values range from −1 to +1, where 1 indicates perfect agreement, 0 indicates chance-level agreement, and negative values indicate less-than-chance agreement (31). For interpretation, the Landis and Koch classification was adopted:<0 = poor; 0–0.20 = slight; 0.21–0.40 = fair; 0.41–0.60 = moderate; 0.61–0.80 = substantial; 0.81–1.00 = almost perfect.

The formula for Fleiss’ kappa is shown in Equation 2:

(2) $$\kappa =\left(\bar{P}-{\bar{P}}_{\text{e}}\right)/\left(1-{\bar{P}}_{\text{e}}\right)$$

Where:

κ is Fleiss’ kappa. $\bar{P}$ is the observed proportion of agreement. ${\bar{P}}_{\text{e}}$ is the expected proportion of agreement by chance.

### 3.3 Kendall’s coefficients

Kendall’s coefficients are originally non-parametric statistical measures utilized to account for the level of association or the degree of agreement between ordinal data. They are particularly advantageous for analyzing datasets or in research studies where the hypotheses required for parametric correlation methods are not fulfilled (32). To account for the ordinal nature of the data, Kendall’s coefficient of concordance (W) and Kendall’s correlation coefficient (τ) were calculated. These tests assess the degree of association among ratings, with greater penalties assigned to misclassifications differing by more than one category compared to adjacent-category errors.

Kendall’s coefficients range from −1 (perfect negative association) to +1 (perfect positive association). For this study,<0.70 = low, 0.70–0.80 = medium, 0.80–0.90 = high, and 0.90–1.00 = very high association (33). The hypotheses were:

H0: No association exists among ratings.
H1: Ratings are associated (i.e., agreement is not due to chance).

Significance was determined at α = 0.05. Kendall’s coefficients (W) is computed as shown in Equation 3:

(3) $$\text{W}=\left(12*\text{S}\right)/\left({\text{k}}^{2}*\text{N}*\left({\text{N}}^{2}-1\right)\right)$$

Where:

• S is the sum of squared deviations of the rank sums.

o First, sum the ranks given by all raters for each subject (patient) to get *R_i_*.

Find the mean of these rank sums, $\bar{R}$. $\text{S}=\sum {\left({\text{R}}_{\text{i}}-\bar{R}\right)}^{2}$• k is the number of raters (e.g., radiotherapists).

• N is the number of subjects (e.g., patients being ranked).

### 3.4 Attribute data considerations

In health preference research (HPR), the identification of attributes and levels may be achieved through exploratory approaches or elicitation techniques, with triangulation of methods recommended to enhance validity. Attributes should be clearly defined and supported with concise descriptions and appropriate visual aids to minimize potential bias. A systematic deconstruction of decision models into attributes and levels is essential to ensure the validity, reliability, and objectivity of HPR study designs. To safeguard validity in the present study, images were evaluated in random order to mitigate sequence bias. Consistent with established standards for attribute agreement testing (34), at least 50 samples covering the full range of process variation were included, each evaluated twice by multiple appraisers (minimum 3–5 clinicians). The unit of analysis for attribute agreement testing is the rated image, not the patient; the 98 ROI and 60 FOV images, each double-scored by 20 raters, exceed established minimum sample-size guidance for kappa-type reliability designs (≥50 items, ≥3–5 raters (35);). Heterogeneity in lesion location across patients was retained by design to assess generalizability of CEA-RT across the anatomical sites where radiation dermatitis is clinically encountered, rather than restricting the estimate to a single body site.

## 4 Results & discussion

### 4.1 Therapists’ absolute agreement

**Figure 4:**
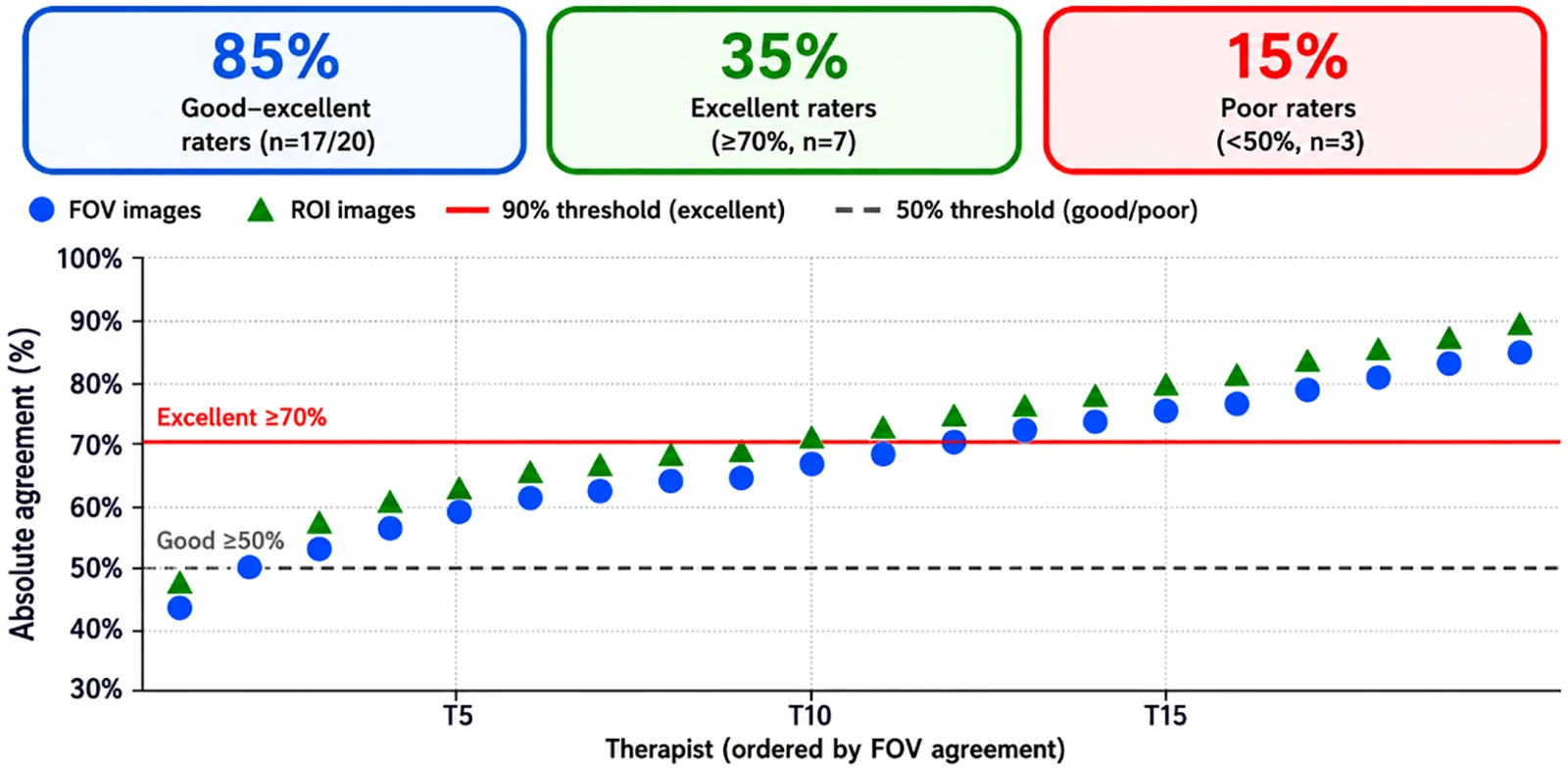
A comparison of intra-rater reliability scores among therapists for assessing radiation-induced erythema using Region of Interest (ROI) and Full-Field-of-View (FOV) images. Intra-rater absolute agreement (%) for each of the 20 participating radiation therapists, shown separately for FOV (blue circles) and ROI (green triangles) image sets. Horizontal reference lines denote the excellent (≥70%) and good/poor threshold (50%). Individual data points replace the aggregated bar chart of the original figure to allow assessment of therapist-level dispersion, skewness, and outliers. Therapists are ordered by ascending FOV agreement.

Intra-rater reliability was assessed for each participating therapist. The percentage of perfect (100%) attribute agreement between the assessment sessions ranged from 45% to 85% for full-field-of-view (FOV) images and from 47% to 88% for region-of-interest (ROI) images. A majority of therapists demonstrated a consistency rate of 45–75% across both image types. When evaluated against a strict match criterion, both FOV and ROI image sets yielded equivalent agreement results. Therapists were categorized based on their agreement rates: 35% (n=7) exhibited excellent agreement (≥70% match), 50% (n=10) demonstrated good agreement (50–69% match), and 15% (n=3) showed poor agreement (<50% match). Overall, 85% of therapists achieved good or excellent intra-rater reliability as illustrated in **Figure 4**. Comparison of grading performance between the ROI and FOV image sets should be interpreted with caution. These two sets differ not only in the number of images (98 ROI vs. 60 FOV) but also in the number of contributing patients (eight for ROI vs. six for FOV) and in the amount of clinical context provided to the grader (isolated ROI vs. full irradiated field with surrounding tissue visible). These structural differences preclude direct statistical comparison of ROI and FOV agreement metrics, and any observed differences may reflect image-set characteristics rather than intrinsic differences in grading difficulty.

### 4.2 Therapists’ kappa test

**Figure 5:**
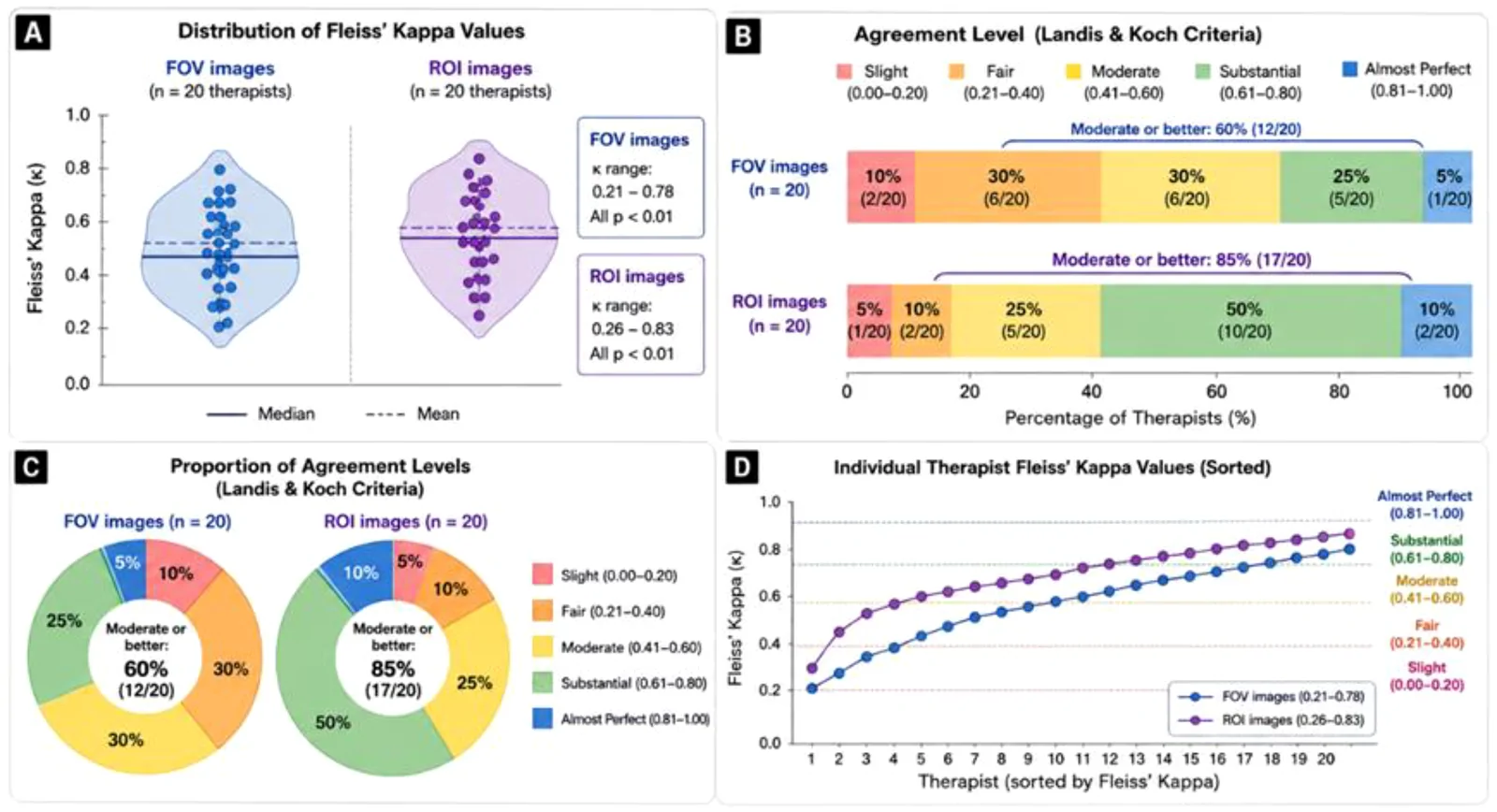
**(A)** Displays the distribution of Fleiss Kappa values for both the FOV and ROI images. **(B)** Exhibits the agreement level using Landis & Koch criterion for all the 20 therapists. **(C)** Shows the proportion of the agreement level between the involved 20 therapists in the study. **(D)** Visualizes the individual therapist Fleiss Kappa values sorted between various degrees of matched agreement. Agreement was generally stronger for ROI images, where 85% of therapists achieved moderate or better self-consistency compared to 60% for FOV images.

To quantify inter-rater agreement, Fleiss’ kappa was calculated, and its statistical significance was evaluated. Applying the Landis and Koch criteria for interpretation, the analysis revealed a range of kappa values from 0.21 to 0.78 for full-field-of-view (FOV) images and 0.26 to 0.83 for region-of-interest (ROI) images, with all results being statistically significant (p< 0.01). Overall, agreement was stronger for ROI images, with 85% of therapists demonstrating moderate or better self-agreement, compared to 60% for FOV images. Substantial or almost perfect agreement was uncommon, observed in a minority of cases. An illustration of kappa test is shown in **Figure 5**.

### 4.3 Therapists’ Kendall’s coefficients

**Figure 6:**
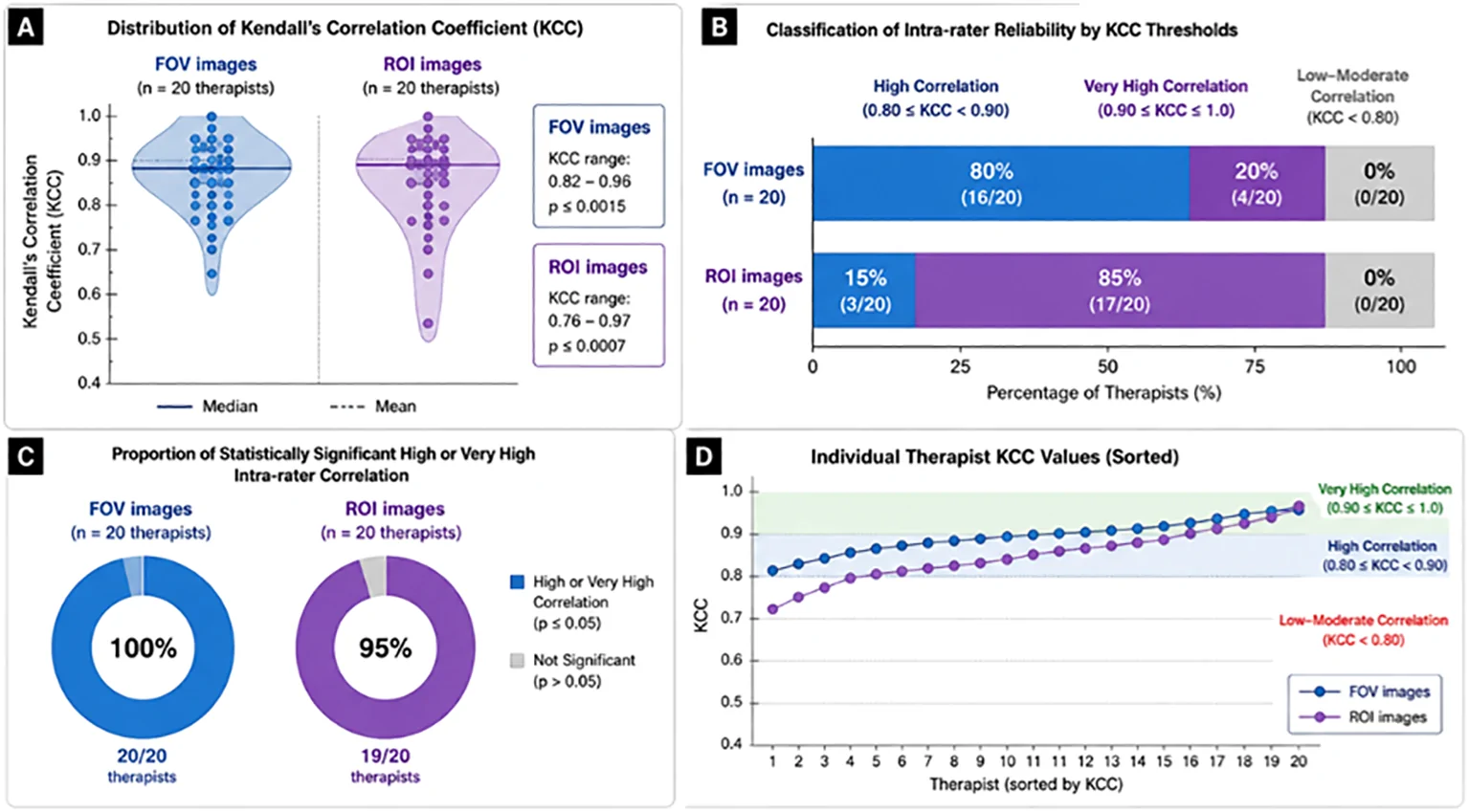
Kendall’s correlation coefficient was used to evaluate intra-rater agreement for ordinal-scale data. **(A)** Displays the distribution of Kendall’s correlation coefficient (KCC) for both the FOV and ROI images. **(B)** Exhibits the classification of the intra-rater reliability by KCC thresholds for all the 20 therapists. **(C)** Shows the proportion of the significance of inter-rater correlation. **(D)** visualizes the individual therapist KCC values sorted between correlation levels. Overall, 95% of ROI and 100% of FOV ratings showed high or very high agreement.

To evaluate agreement while accommodating the ordinal characteristics of the data, Kendall’s correlation coefficient (KCC) was employed. Intra-rater reliability analysis demonstrated strong correlations, with KCC values ranging from 0.82 to 0.96 for FOV images (p ≤ 0.0015) and from 0.76 to 0.97 for ROI images (p ≤ 0.0007). High correlation (0.80 ≤ KCC< 0.90) was observed among 80% (16/20) of therapists for FOV images and 10% (2/20) for ROI images, while very high correlation (0.90 ≤ KCC ≤ 1.0) was found in 20% (4/20) and 85% (17/20) of therapists, respectively. Overall, all FOV images (100%) and the majority of ROI images (95%) demonstrated statistically significant high or very high intra-rater correlation. The results of Kendall’s coefficients are visually displayed in **Figure 6**.

### 4.4 Therapists attribute data considerations

**Figure 7:**
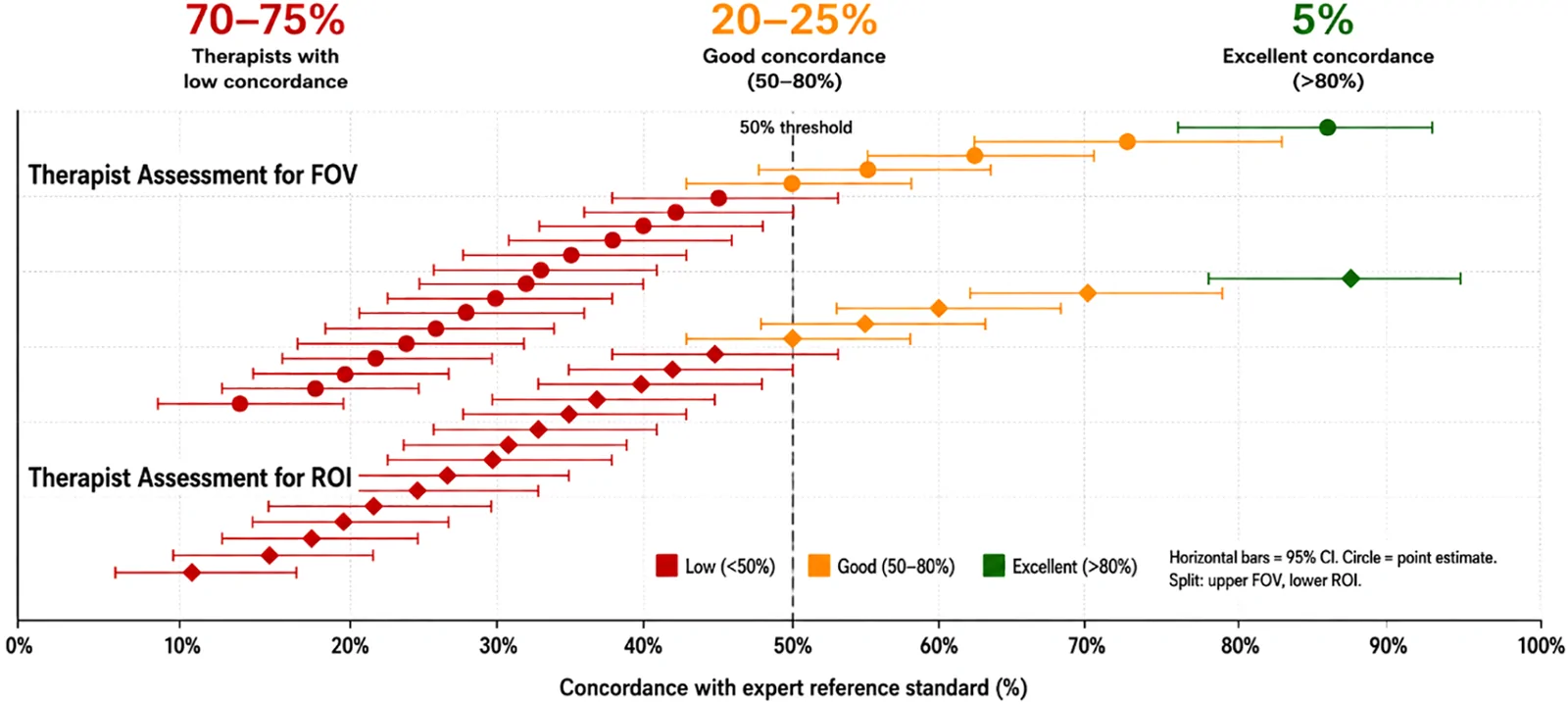
Agreement between therapists’ ratings and the standard ranged from 13–85% for FOV images and 10–88% for ROI images. Forest-plot representation of each therapist’s concordance with the single-expert reference standard, presented with 95% confidence intervals for both FOV (upper) and ROI (lower) image sets. The vertical dashed line at 50% demarcates the low/good threshold. Color of each point and interval encodes the performance category.

The concordance between each therapist’s ratings and the reference standard was evaluated using 100% agreement analysis. As illustrated in **Figure 7**, forest plot representation, the 95% confidence intervals revealed that most therapists provided correct ratings in 20–65% of cases. Agreement with the standard ranged from 13% to 85% for FOV images and from 10% to 88% for ROI images. Therapists were subsequently grouped into performance categories: less than 50% agreement was classified as low, 50–80% as good, and greater than 80% as excellent. For FOV images, 70% (14/20) of therapists demonstrated low agreement, 25% (5/20) achieved good agreement, and 5% (1/20) attained excellent agreement. The results were similar for ROI images, with 75% (15/20) showing low agreement, 20% (4/20) good, and 5% (1/20) excellent. Overall, 70-75% of therapists demonstrated low agreement with the standard, while 25-30% demonstrated good to excellent agreement.

### 4.5 Study limitations

A primary limitation of this study is the use of a single expert radiation therapist as the reference standard. Although the selected evaluator possessed extensive experience in assessing radiation-induced erythema and was highly familiar with the CEA-RT grading criteria, reliance on a single assessor may introduce subjective bias and does not account for variability among expert observers. Photographic assessment is an established clinical modality with documented validity against in-person examination (pooled κ = 0.67–0.77 across 44 teledermatology studies; Bourkas et al., 2023); nonetheless, tactile findings such as infiltration, edema, and temperature are not captured by any image-based method. Future validation studies should consider establishing a consensus-based reference standard using expert panels comprising radiation oncologists and experienced radiation therapists. Additionally, objective optical assessment techniques, including colorimetry, diffuse reflectance spectroscopy, and hyperspectral imaging, may provide quantitative measures of erythema severity that could complement visual grading and improve the validity of the reference standard.

The relatively limited sample size represents another important study limitation. Although twenty radiation therapists participated and multiple repeated assessments were obtained, the image dataset originated from only nine patients. Consequently, the reported agreement statistics should be interpreted cautiously, as smaller sample sizes may increase uncertainty in reliability estimates and limit statistical generalizability. Furthermore, the single-center design may not fully represent variability in clinical practices across different institutions. Future multicenter studies involving larger cohorts of clinicians and patients are necessary to provide more precise estimates of inter-rater agreement and to establish the psychometric robustness of the CEA-RT scale across diverse clinical environments.

The study population consisted exclusively of Caucasian patients, limiting the generalizability of the findings to individuals with darker skin phototypes. This limitation is particularly important because the visual identification of erythema may become increasingly difficult as baseline skin pigmentation increases, potentially affecting grading accuracy and inter-observer agreement. Consequently, the reliability characteristics reported in this study may not be directly transferable to more diverse patient populations. Future validation studies should intentionally recruit participants representing a broad spectrum of Fitzpatrick skin types to determine whether modifications to the CEA-RT criteria or supplemental objective imaging techniques are required to maintain diagnostic accuracy across different pigmentation levels. These limitations are intrinsic to any visual grading instrument, including the widely used RTOG and CTCAE scales; CEA-RT does not claim to resolve them, but to provide standardized radiation-specific terminology and finer early-erythema resolution within that shared constraint.

### 4.6 Patient-reported outcomes and the limits of clinician assessment

An important dimension not addressed in the present study is the potential divergence between clinician-assessed erythema severity and patient-reported experience of skin toxicity (36). Emerging evidence demonstrates that clinicians systematically under-report dermatitis symptom severity relative to patients’ own assessments, suggesting that visual grading tools such as CEA-RT, however reliably applied, may not fully capture the patient experience of radiation dermatitis. Future investigations should pair CEA-RT grading with validated patient-reported outcome measures — such as the Skindex-16, the FACT-G, or purpose-built radiation dermatitis patient-reported outcome instruments — to provide a more comprehensive and patient-centered assessment of treatment-related skin toxicity.

## 5 Conclusions

The evaluation of the CEA-RT scale revealed a distinct disparity between its reliability and its accuracy. Intra-rater reliability was strong, with 85% of therapists achieving “good” or better self-agreement and grade differences within ±1 for ≥98% of images. However, inter-rater reliability was markedly lower, with Fleiss’ κ indicating only overall “fair” agreement among all therapists. When evaluated against a reference standard, the overall accuracy was moderate. Concordance with the standard was statistically significant and moderate or better for 50–60% of therapists; however, only 25–30% demonstrated good to excellent agreement. This retrospective, image-based inter-rater and intra-rater reliability study represents an early and exploratory validation step for the CEA-RT scale, and the findings should not be interpreted as definitive evidence of clinical utility or readiness for routine deployment. This indicates that while a majority of assessments were satisfactory, a substantial proportion failed to achieve a high degree of fidelity to the benchmark.

These findings are constrained by limitations, including the use of digital photographs—which may introduce technical variability—and a sample limited to Caucasian skin tones, affecting generalizability. Therefore, while therapists can apply the CEA-RT scale consistently with themselves, its accuracy for objective measurement is moderate. Future validation should involve *in vivo* studies with a diverse patient population and consider supplemental training to improve concordance. In addition, it is compulsory to acknowledge that This study was retrospective in design, based on a pre-existing anonymized image dataset. Consequently, clinical variables including formal performance status, relevant comorbidities, concurrent or prior systemic therapy, and standardized skin-care or prophylactic measures were not systematically captured. The absence of these data represents a limitation and should be addressed in prospective designs, where such variables should be recorded and reported as potential confounders of erythema severity grading.

Future prospective studies should incorporate serial on-treatment assessment conducted *in vivo*, standardized and reproducible image acquisition protocols, clinically diverse patient cohorts with varied skin phototypes (Fitzpatrick Types I–VI), and patient-reported outcome measures for erythema-associated symptoms. Multi-rater consensus grading or objective colorimetric reference standards would provide a substantially higher level of evidence before broad clinical adoption of CEA-RT can be recommended.

## Data Availability

The raw data supporting the conclusions of this article will be made available by the authors, without undue reservation.
